# Heterogeneous Responses of Gastric Cancer Cell Lines to Tenovin-6 and Synergistic Effect with Chloroquine

**DOI:** 10.3390/cancers12020365

**Published:** 2020-02-05

**Authors:** Xiangyu Ke, Qingsong Qin, Tianyi Deng, Yueyan Liao, Shou-Jiang Gao

**Affiliations:** 1 Laboratory of Human Virology and Oncology, Shantou University Medical College, Shantou 515000, China; 17xyke@stu.edu.cn (X.K.); 17tydeng@stu.edu.cn (T.D.); 17yyliao@stu.edu.cn (Y.L.); 2 UPMC Hillman Cancer Center, Department of Microbiology and Molecular Genetics, University of Pittsburgh, Pittsburgh, PA 15213, USA

**Keywords:** Gastric cancer, Epstein–Barr virus (EBV), Tenovin-6, chloroquine, autophagy, p53 activation

## Abstract

Gastric cancer (GC) is the fifth most frequently diagnosed cancer and the third leading cause of cancer death. Approximately 15% of GC is associated with Epstein–Barr virus (EBV). GC is largely incurable with a dismal five-year survival rate. There is an urgent need to identify new therapeutic agents for the treatment of GC. Tenovin-6 was initially identified as a p53 activator, but it was later found to inhibit autophagy flux, and the protein deacetylase activity of sirtuins. Tenovin-6 shows promising therapeutic effect in various malignancies. However, it remains unknown whether Tenovin-6 is effective for GC. In this study, we found that EBV-positive and -negative GC cell lines were sensitive to Tenovin-6 but with different response times and doses. Tenovin-6 suppressed anchorage-independent growth of GC cells. Tenovin-6 induced different levels of apoptosis and phases of cell-cycle arrest depending on the cell lines with some manifesting gap 1 (G1) and others showing synthesis (S) phase cell-cycle arrest. Mechanistically, Tenovin-6 induced autophagy or p53 activation in GC cells depending on the status of *TP53* gene. However, initiation of autophagy following treatment with Tenovin-6 conferred some protective effect on numerous cells. Combined treatment with Tenovin-6 and autophagy inhibitor chloroquine increased the cytotoxic effect by inducing microtubule-associated protein 1 light chain 3B (LC3B)-II accumulation, and by enhancing apoptosis and cell-cycle arrest. These results indicated that Tenovin-6 can be used as a potential therapeutic agent for GC, but the genetic background of the cancer cells might determine the response and mechanism of action. Treatment with Tenovin-6 alone or in combination with chloroquine could be a promising therapeutic approach for GC.

## 1. Introduction

Gastric cancer (GC) is the fifth most frequently diagnosed cancer and the third leading cause of cancer death. In 2018, there were 1,000,000 new cases and an estimated 783,000 deaths of GC [1]. Approximately 15% of GC is associated with Epstein–Barr virus (EBV) [2]. Early gastric cancer remains asymptomatic and lacks effective biomarkers for detection. Thus, most gastric cancer cases are diagnosed with distant metastasis at an advanced stage [3]. Advanced GC is largely incurable with a dismal five-year survival rate. Cisplatin and 5-fluorouracil remain the mainstay of treatment for people with advanced GC, both of which have high side effects [4]. There is an urgent need to explore new therapeutic agents for the treatment of GC.

Tenovin-6 was initially identified as a p53 activator, but it was later found to inhibit the protein deacetylase activity of metabolic sensors sirtuin 1 (SIRT1), SIRT2, and SIRT3 [5,6]. Numerous studies showed that Tenovin-6 initiates but subsequently impairs the autophagy flux by increasing the levels of microtubule-associated protein 1 light chain 3B (LC3B)-II and/or sequestosome-1 (SQSTM1)/p62 in diverse cell types [7,8,9,10]. Tenovin-6 was shown to have a promising anti-neoplastic effect in vitro and in vivo in various malignancies [6,7,8,11,12,13]. Tenovin-6 upregulated the death receptor 5 and enhanced the cytotoxic effects of 5-fluorouracil and oxaliplatin in colon cancer cells [14]. In gastric cell lines, Tenonvin-6 manifested an inhibitory effect on cell survival in part by upregulating the expression of death receptor 5 [13]. However, this effect was not detected in all the cell lines examined. Because of the diverse genetic heterogeneities of GC including the status of the *TP53* gene and the presence of EBV infection in a subset of gastric cancer, it remains essential to further evaluate the therapeutic effect of Tenovin-6 for GC. In particular, whether initiation and impairment of the autophagy flux by Tenovin-6 is universal in GC cell lines, which could explain its inhibitory effect, remains unclear.

Chloroquine was initially used as an antimalarial drug, but it was later shown to be an effective anticancer drug [15,16]. Autophagy is an evolutionarily conserved cellular homeostatic process that is responsible for degrading damaged proteins or unnecessary cellular organelles and proteins [17]. The anticancer effect of chloroquine may partially be due to its inhibitory action on autophagy. Accumulating evidence indicates that chloroquine can sensitize cancer cells to radiation and other anticancer drugs [16]. Recent studies indicate that autophagy inhibition could enhance the efficacy of antitumor drugs in cancer therapy [18,19].

In this study, we demonstrated that numerous EBV-positive and -negative GC cell lines were sensitive to Tenovin-6 but with different response times and doses. Tenovin-6 suppressed anchorage-independent growth of GC cells. Tenovin-6 induced cell-cycle arrest and apoptosis depending on the cell lines with some manifesting gap 1 (G1) or synthesis (S) phase cell-cycle arrest and others showing apoptosis. Mechanistically, Tenovin-6 induced autophagy or p53 activation in GC cells depending on the genetic background. Initiation of autophagy following treatment with Tenovin-6 conferred some protective effect on numerous cells; however, combined treatment of Tenovin-6 and chloroquine increased the cytotoxic effect of Tenovin-6 by inducing LC3B-II accumulation, and by enhancing apoptosis and G1 cell-cycle arrest. These results indicate that Tenovin-6 could be a potential therapeutic agent for GC but the genetic background of the cancer cells might determine their response and mechanism of action. Treatment with Tenovin-6 alone or in combination with chloroquine could be a promising therapeutic approach for GC.

## 2. Results

### 2.1. Tenovin-6 Inhibits Cell Proliferation and Anchorage-Independent Growth of GC Cells

**Figure 1:**
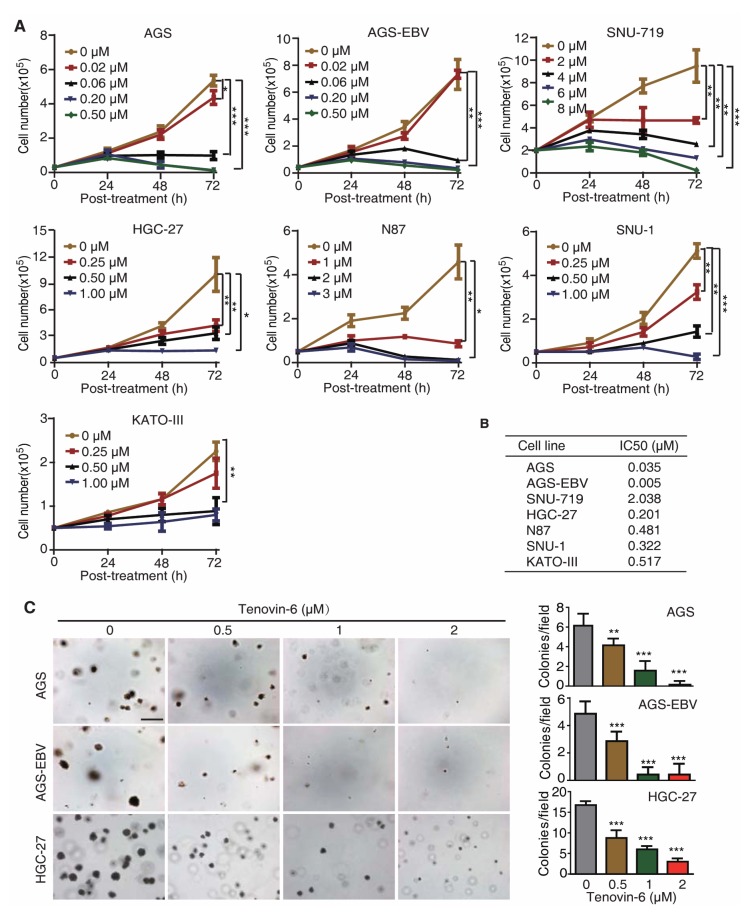
Tenovin-6 inhibits cell proliferation and anchorage-independent growth of gastric cancer (GC) cells. (**A**) Examination of cell proliferation following treatment with Tenovin-6. Cells seeded at 2.5 × 10^4^ or 5 × 10^4^ cells/well were treated with the indicated concentrations of Tenovin-6 and counted at 24, 48, and 72 h post treatment. * *p* < 0.05, ** *p* < 0.01, *** *p* < 0.001. (**B**) The half maximal inhibitory concentration (IC_50_) values were calculated using SPSS software based on the relative cell numbers at 72 h post treatment in all GC cell lines. (**C**) Suppression of anchorage-independent growth of AGS, AGS-EBV, and HGC-27 cells by Tenovin-6. Representative pictures captured at 5× magnification are presented in the left panel. Colonies with diameter >50 μm were counted, and colony numbers in each field are presented in the right panel. * *p* < 0.05, ** *p* < 0.01, *** *p* < 0.001. Scale bar: 500 μm.

To test whether Tenovin-6 had a universal inhibitory effect on GC cells, we treated seven gastric cancer cell lines with different concentrations of Tenovin-6, including EBV-positive cell lines AGS-EBV and SNU-719, and EBV-negative cell lines AGS, HGC-27, N87, SNU-1, and KATO-III. AGS-EBV cells were obtained by infecting AGS cells with a recombinant EBV M81 [20], while SNU-719 cells was isolated from a GC patient [21,22]. Tenovin-6 potently inhibited cell proliferation in a dose- and time-dependent manner in all seven cell lines examined (Figure 1A); however, the sensitivities of these cell lines to Tenovin-6 varied. We calculated the half maximal inhibitory concentration (IC_50_) value to Tenovin-6 for each cell line at 72 h post treatment (Figure 1B). AGS and AGS-EBV cells were the most sensitive lines with IC_50_ values of 0.035 and 0.005 μmol/L, respectively, followed by HGC-27, SNU-1, N87, and KATO-III cells with IC_50_ values of 0.201, 0.322, 0.481, and 0.517 μmol/L, respectively (Figure 1B). SNU-719 cells were the least sensitive to Tenovin-6 with an IC_50_ value of 2.038 μmol/L (Figure 1B).

Of the seven GC cell lines, AGS, AGS-EBV, and HGC-27 cells were capable of anchorage-independent growth in soft agar. Tenovin-6 treatment significantly reduced the anchorage-independent growth efficiencies of AGS, AGS-EBV, and HGC-27 cells (Figure 1C).

Together, these results indicated that GC cell lines were susceptible to Tenovin-6, and the sensitivity of GC cell lines to Tenovin-6 depended on their genetic background rather than EBV status of these cell lines.

### 2.2. Tenovin-6 Induces Apoptosis and Cell-Cycle Arrest of GC Cells

**Figure 2:**
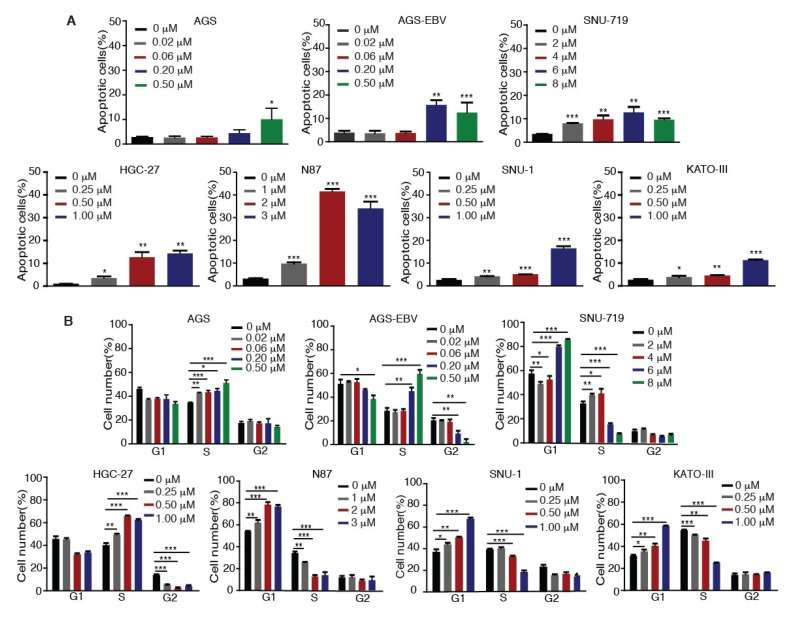
Tenovin-6 induces apoptosis and cell-cycle arrest of GC cells. (**A**) Percentages of apoptotic cells in the indicated cell lines after Tenovin-6 treatment based on annexin V and propidium iodide (PI) staining. * *p* < 0.05, ** *p* < 0.01, *** *p* < 0.001. (**B**) Percentages of cells at gap 1 (G1), synthesis (S), and G2/mitotic (M) phases of the indicated cell lines after Tenovin-6 treatment based on PI staining. * *p* < 0.05, ** *p* < 0.01, *** *p* < 0.001.

We investigated whether Tenovin-6 inhibition of proliferation of GC cells was due to the induction of apoptosis. GC cells were treated with Tenovin-6 for 48 h except for HGC-27 cells, which were treated for 72 h to give an obvious cytotoxic effect, and they were then examined for apoptosis by annexin V and propidium iodide (AV/PI) staining. Tenovin-6 induced apoptosis in GC cells in a dose-dependent manner in all seven cell lines (Figure 2A). At higher doses of Tenovin-6, apoptosis was observed in 10%–15% of cells in most of the cell lines except for N87, which had over 30% apoptotic cells (Figure 2A). Although the highest concentration of Tenovin-6 was used for SNU-719 cells, these cells did not have a higher number of apoptotic cells compared to other cell lines, indicating that SNU-719 cells were the most resistant to Tenovin-6 (Figure 2A).

We further investigated whether Tenovin-6 induced cell-cycle arrest. GC cells were treated with Tenovin-6 for 48 h except for HGC-27 cells, which were treated for 72 h. Tenovin-6 significantly increased the numbers of cells in G1 phase and decreased the numbers of cells in S phase in a dose-dependent manner in N87, SNU-1, and KATO-III cells (Figure 2B). In contrast, the numbers of cells in S phase were increased, while the numbers of cells in G1 and G2 phases were decreased in a dose-dependent manner in AGS, AGS-EBV, and HGC-27 cells by Tenovin-6 (Figure 2B). Interestingly, Tenovin-6 increased the numbers of cells in S phase and decreased the numbers of cells in G1 and G2 phases at lower doses (2 and 4 μM) in SNU-719 cells; however, at higher doses (6 and 8 μM), Tenovin-6 increased the numbers of cells in G1 phase and decreased the numbers of cells in S phase (Figure 2B).

These results indicated that Tenovin-6 inhibited cell proliferation by inducing apoptosis and cell-cycle arrest; however, the responses of different cell lines to Tenovin-6 varied with some manifesting G0/G1 or S phase cell-cycle arrest and others showing apoptosis; the results also indicated that these differential responses were not dependent on the EBV status.

### 2.3. Tenovin-6 Initiates But Blocks Autophagy Flux in Some GC Cell Lines

**Figure 3:**
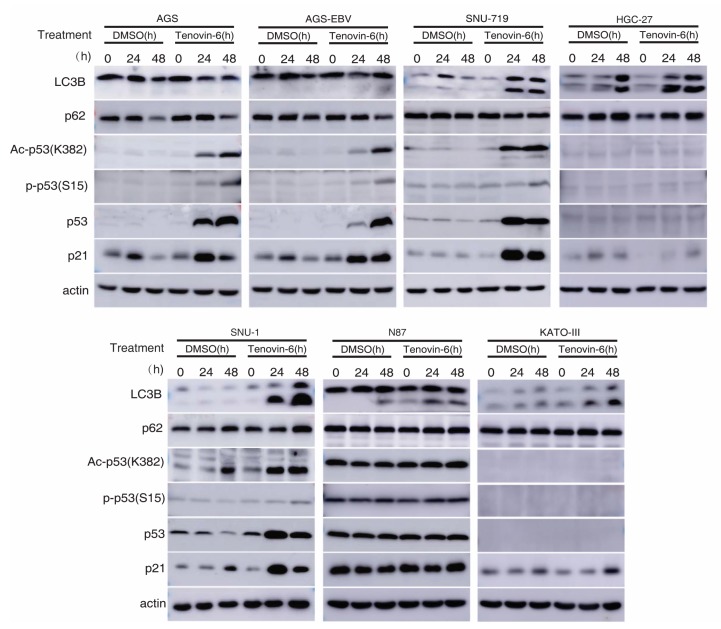
Tenovin-6 induces p53 activation or initiated but failed to induce full program of autophagy in GC cells. The levels of microtubule-associated protein 1 light chain 3B (LC3B), sequestosome-1 (SQSTM1)/p62 (p62), acetylated-p53 (Ac-p53 (K382)), phospho-p53 (Ser15) (P-p53 (S15)), p53, and p21 were examined in GC cell lines following treatment with Tenovin-6 for 24 and 48 h. Tenovin-6 was used at 0.5 μM for AGS, AGS-EBV, and SNU-1 cells, 6 μM for SNU-719 cells, 1 μM for HGC-27 and KATO-III cells, and 2 μM for N87 cells.

Autophagy is marked by the early induction of LC3B-II protein followed by subsequent degradation of p62 protein, while the inhibition of autophagy leads to p62 protein accumulation [23,24]. Tenovin-6 was shown to initiate autophagy by inducing LC3B-II protein in diverse cell types; however, the subsequent autophagy flux is blocked by Tenovin-6, resulting in unchanged or increased level of p62 [7,8,9,10]. We examined LC3B-II and p62 protein levels in GC cell lines following Tenovin-6 treatment. Tenovin-6 had no effect on LC3B-II and p62 protein levels in AGS and AGS-EBV cells, indicating that Tenovin-6 did not initiate or block autophagy in these cells (Figure 3 and Supplementary Figure S1). In contrast, Tenovin-6 treatment increased the level of LC3B-II protein in SNU-719, HGC-27, SNU-1, N87, and KATO-III cells; however, p62 protein level had either no change or a slight increase, indicating that Tenovin-6 blocked the autophagy flux in these cells (Figure 3).

### 2.4. Tenovin-6 Induces and Activates p53 in Some GC Cell Lines

Tenovin-6 was initially identified as a p53 activator [5,6], and p53 regulates the autophagic pathway, as well as cell cycle and apoptosis [25,26]. We examined p53 activation in GC cells. Among all the seven cell lines, the *TP53* gene is wild-type in AGS, AGS-EBV, SNU-719, and SNU-1 cells, is mutated in HGC-27 and N87 cells, and is null in KATO-III cells (Table 1) [22,27,28,29,30,31,32]. In cell lines with wild-type *TP53* gene, Tenovin-6 induced higher total and acetylated p53 (ac-p53) levels (Figure 3). However, robust phosphorylated p53 (p-p53) levels were only observed in AGS and AGS-EBV cells, while SNU-719 and SNU-1 cells only had weak increases in p-p53 levels (Figure 3). In cell lines with mutated or null *TP53* gene, there was either no increase (N87) or no detectable (HGC-27 and KATO-III) p53, ac-p53, and p-p53 (Figure 3). In agreement with these results, Tenovin-6 increased the levels of p21 protein in AGS, AGS-EBV, SNU-719, and SNU-1 cells, indicating that all four cell lines had p53 activation; however, no increase of p21 protein was observed in HGC-27, N87, and KATO-III cells (Figure 3). By comparing the effect of Tenovin-6 on AGS and AGS-EBV cells, we did not observe any differences in autophagic response and p53 activation (Figure 3). Overall, we did not observe any correlation between autophagy initiation or the blockage of autophagy flux and p53 activation following Tenovin-6 treatment in these cell lines.

Taken together, we concluded that individual GC cell lines had different responses to Tenovin-6 treatment with some manifesting p53 activation and others showing autophagy initiation and blockage of autophagy flux, which might explain their different responses in apoptosis and cell-cycle arrest (Table 1). In addition, none of these responses were correlated with the EBV status of the cell lines. Nevertheless, cell lines that had initiation and blockage of autophagy flux following treatment with Tenovin-6 (SNU-719, SNU-1, HGC-27, N87, and KATO-III cells) appeared to be protected from Tenovin-6 and, hence, were more resistant to Tenovin-6, regardless of their *TP53* gene status (Table 1). For cell lines that did not have initiation and blockage of autophagy flux following treatment with Tenovin-6 (AGS and AGS-EBV cells), they were sensitive to Tenovin-6 and had high levels of p53 activation giving their wild-type *TP53* gene status (Figure 3).

### 2.5. Tenovin-6 and Chloroquine Synergistically Inhibit Cell Proliferation in GC Cells

**Figure 4:**
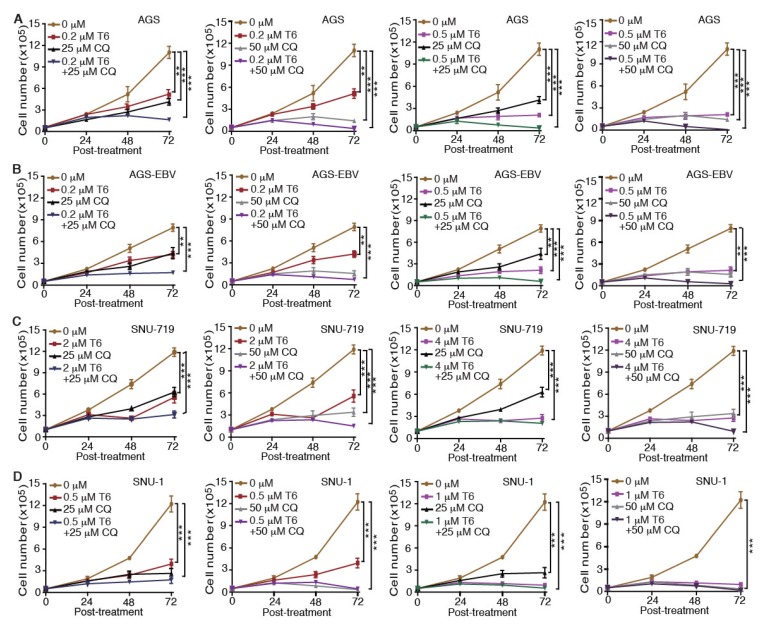
Chloroquine (CQ) enhances the inhibitory effect of Tenovin-6 (T6) on cell proliferation in GC cells. (**A**–**D**) Examination of cell proliferation in AGS (**A**), AGS-EBV (**B**), SNU-719 (**C**), and SNU-1 (**D**) cells following treatment with different concentrations of T6 or CQ alone, or in combination. * *p* < 0.05, ** *p* < 0.01, *** *p* < 0.001.

**Figure 5:**
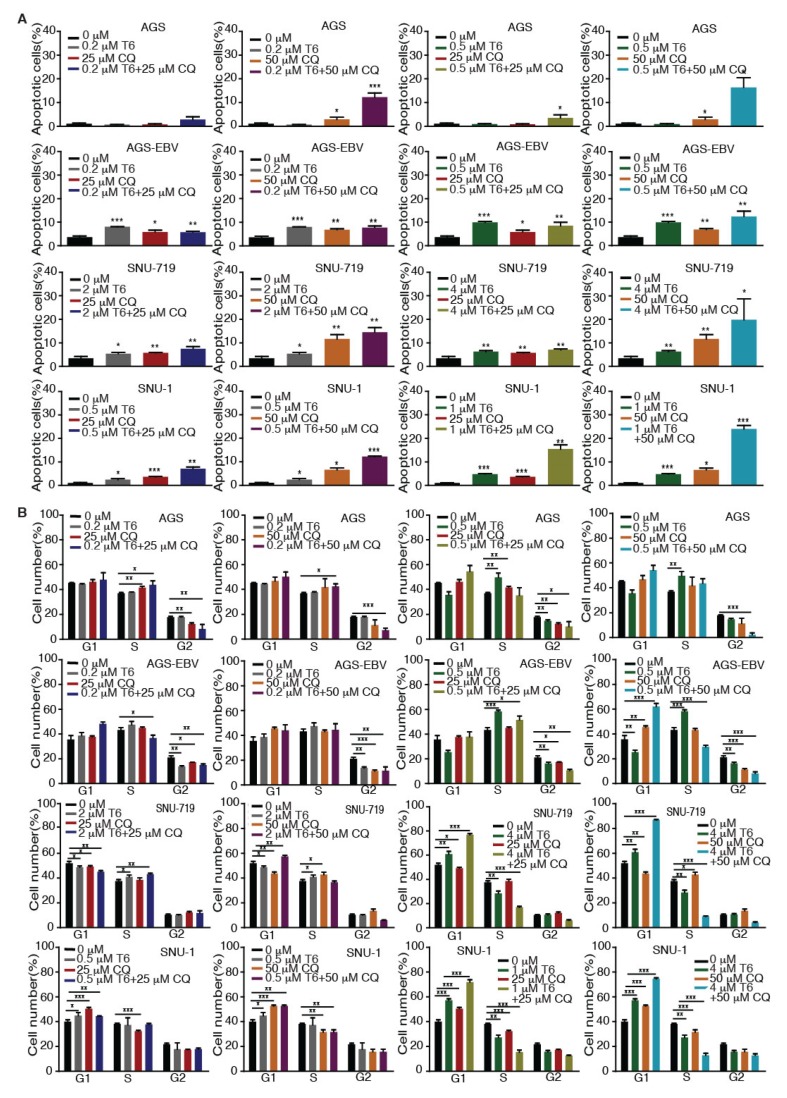
Tenovin-6 (T6) and chloroquine (CQ) synergistically induce apoptosis and cell-cycle arrest in GC cells. (**A**) Examination of apoptotic cells by annexin V and propidium iodide (PI) staining in AGS, AGS-EBV, SNU-719, and SNU-1 cells following treatment with different concentrations of T6 or CQ alone or in combination. * *p* < 0.05, ** *p* < 0.01, *** *p* < 0.001. (**B**) Examination of cell-cycle phases in AGS, AGS-EBV, SNU-719, and SNU-1 cells following treatment with different concentrations of T6 or CQ alone or in combination. * *p* < 0.05, ** *p* < 0.01, *** *p* < 0.001.

Recent studies revealed that autophagy has a cytoprotective effect, and that inhibition of autophagy could enhance the efficacy of anti-tumor drugs in cancer therapy [18,19,33]. Our results showed that Tenovin-6 initiated and then blocked the autophagy flux in SNU-719, HGC-27, N87, SNU-1, and KATO-III cells, which could be the reason for their relative resistance to Tenovin-6 compared to AGS and AGS-EBV, which did not have any autophagy responses (Figure 1 and Figure 3). Therefore, we explored if combined treatment with Tenovin-6 with another inhibitor chloroquine (CQ), a lysosome inhibitor blocking autophagy flux, could enhance the cytotoxic effect of Tenovin-6. We examined the combined effect of the two inhibitors on AGS and AGS-EBV cells that did not have any autophagy responses, and SNU-719 and SNU-1 cells that had autophagy responses (Figure 3). SNU-719 was also the least responsive cell line among all the GC cell lines examined (Figure 1A,B) and SNU-1 cells had the least induction of p-p53 following Tenovin-6 treatment (Figure 3). Chloroquine alone inhibited the proliferation of AGS, AGS-EBV, SNU-719, and SNU-1 cells as effectively as Tenovin-6 did (Figure 4), confirming that blockage of autophagy was essential for induction of cytotoxicity. Interestingly, combined treatment with both inhibitors further enhanced the cytotoxicity in all four cell lines (Figure 4). In fact, the inhibitory effect of combined treatment with lower doses of Tenovin-6 (0.2 μM) and chloroquine (25 μM) was at least as effective as a higher dose of single treatment of either Tenovin-6 (0.5 μM) or chloroquine (50 μM) (Figure 4). In agreement with these results, combined treatment with Tenovin-6 and chloroquine increased the numbers of apoptotic cells in all four cell lines (Figure 5A). While Tenovin-6 only induced S phase arrest but not G1 phase arrest in AGS and AGS-EBV cells (Figure 2B), combined treatment with Tenovin-6 and chloroquine led to G1 phase arrest in these cells (Figure 5B). Tenovin-6 alone was sufficient to induce G1 phase arrest in SNU-719 and SNU-1 cells (Figure 2B). Interestingly, combined treatment with both inhibitors further increased G1 phase arrest in these cells (Figure 5B). Thus, increased G1 phase arrest appeared to be essential for the cytotoxic and synergistic effects of the combined treatment.

**Figure 6:**
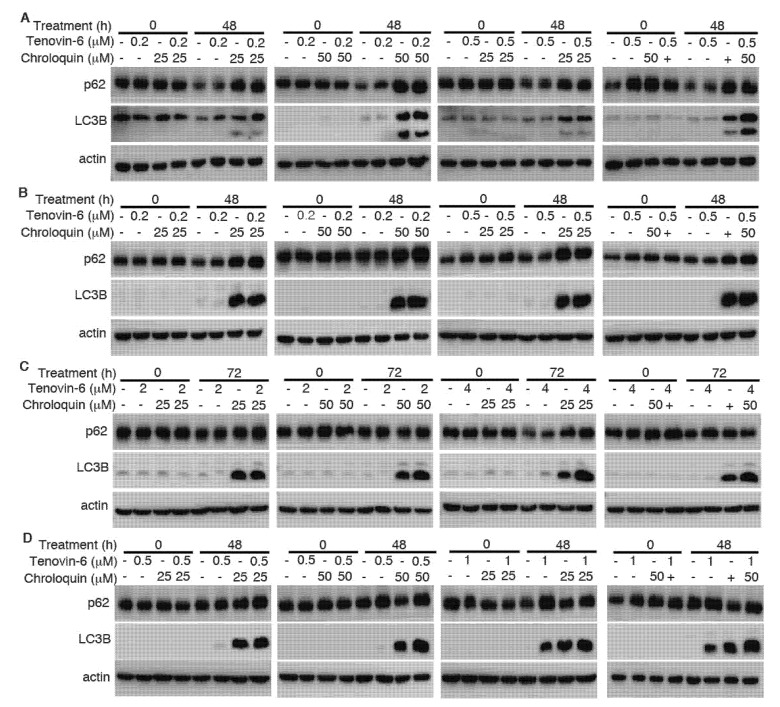
Chloroquine induces microtubule-associated protein 1 light chain 3B (LC3B)-II but blocks autophagy flux to enhance cytotoxicity of Tenovin-6. (**A**–**D**) Examination of LC3B and sequestosome-1 (SQSTM1)/p62 (p62) protein levels following treatment with Tenovin-6 or chloroquine alone, or in combination for 48 h in AGS (**A**), AGS-EBV (**B**), SNU-719 (C), and SNU-1 (**D**) cells.

To determine whether the enhanced cytotoxic effect of Tenovin-6 by chloroquine was due to the inhibitory effect of chloroquine on autophagy flux, we examined LC3B-II and p62 protein levels. While Tenovin-6 did not alter LC3B-II and p62 protein levels in AGS and AGS-EBV cells, chloroquine alone or in combination with Tenovin-6 indeed effectively induced LC3B-II protein in these cells; however, there was no change of p62 protein level, indicating the inhibition of autophagy flux, which likely contributed to the increased apoptosis and induction of G1 cell cycle arrest in these cells (Figure 6A,B and Supplementary Figure S1). While Tenovin-6 alone induced LC3B-II protein in SNU-719 and SNU-1 cells, chloroquine alone or in combination with Tenovin-6 further increased a higher level of LC3B-II protein; however, chloroquine or Tenovin-6 alone, or in combination did not alter the level of p62 protein, which might contribute to the enhanced cytotoxic effect of Tenovin-6 by chloroquine in these cells (Figure 6C,D).

Together, these results indicated that the combined treatment of Tenovin-6 and chloroquine could effectively inhibit cell proliferation, and it synergistically induced apoptosis and cell-cycle arrest in different GC cell lines, including those with and without EBV infection. The autophagy inhibitory effect of chloroquine likely contributed to these synergistic effects.

## 3. Discussion

Epstein–Barr virus (EBV) is closely associated with several types of malignancies, including Burkitt lymphoma (BL), Hodgkin’s lymphoma (HL), nasopharyngeal carcinoma (NPC), and gastric cancer (GC) [34,35,36]. In different EBV-associated malignancies, the virus manifests distinct latency patterns, namely, type I, II, III, or Wp-restricted latency [35,36,37]. EBV-associated GC (EBVaGC) belongs to latency type I or II, expressing *EBERs*, *EBNA-1*, *BARTs*, and BART microRNAs (miRNAs), respectively [38,39]. Approximately half of EBVaGC cases also express *LMP-2A* [38,39]. In the present study, we compared the effect of Tenovin-6 on EBV-negative GC cell lines AGS, HGC-27, N87, SNU-1, and KATO-III, and EBV-positive GC cell lines AGS-EBV and SNU-719. All the GC cell lines were sensitive to Tenovin-6; however, SNU-719 was clearly the least sensitive cell line (IC_50_ value of 2.038 μmol/L for SNU-719), while the AGS and AGS-EBV cells were the most sensitive cell lines (0.005 and 0.035 μmol/L) (Figure 1A,B). A previous study determined that the IC_50_ values of six EBV-negative GC cell lines to Tenovin-6 were between 2.34 and 4.28 μmol/L [13]. While comparisons among cell lines were impossible because of their different genetic backgrounds, AGS and AGS-EBV had the same genetic background, thereby allowing assessing the effect of EBV. Surprisingly, AGS-EBV cells were more sensitive to Tenovin-6 than AGS cells, suggesting that the high resistance of SNU-719, an EBV-positive cell line, to Tenovin-6 was unlikely due to its EBV status.

One of the most commonly mutated genes in human cancer is tumor suppressor gene *TP53* [40]. Approximately 50% of GCs have a *TP53* gene mutation [41]. These mutations disrupt the transcriptional activation ability of p53 protein, including induction of genes associated with cell cycle, apoptosis, and DNA repair [42]. Among the seven cell lines examined in this study, four have wild-type *TP53* (AGS, AGS-EBV, SNU-719 and SNU-1), two have mutated *TP53* (HGC-27 and N87), and one has null TP53 (KATO-III) [22,27,28,29,30,31,32]. However, we did not observe any correlation of the sensitivity of these cell lines to Tenovin-6 with the status of *TP53* gene alone.

We analyzed the potential mechanism of Tenovin-6 inhibition of cell proliferation in GC cells. It was reported that GC cells are sensitive to both apoptotic and autophagic cell death [43,44]. We found that the responses of different cell lines to Tenovin-6 varied with some manifesting G1 or S phase cell-cycle arrest and others showing apoptosis, and that these differential responses were not depend on the EBV status (Figure 2). Because Tenovin-6 was shown to initiate autophagy by inducing LC3B-II protein in diverse cell types, we examined its effect on LC3B-II and p62 protein levels in GC cell lines. Tenovin-6 was initially identified as a p53 activator [5,6], and p53 regulates the autophagic pathway as well as cell cycle and apoptosis [25,26]. Hence, we also examined p53 activation in GC cells. We found that p53 activation following Tenovin-6 treatment completely depended on the genetic status of the *TP53* gene. p53 activation was detected in all four cell lines with wild-type *TP53* gene. However, no p53 activation was detected in the three cell lines with either mutated or null *TP53* gene (Figure 3). Interestingly, we did not find any correlation of p53 activation with autophagy initiation (Figure 3), which might explain the different responses in apoptosis and cell-cycle arrest of these cell lines (Table 1). In addition, none of these responses were correlated with the EBV status of the cell lines.

Induction of autophagy can either be a pro-survival mechanism protecting cells against stress-induced killing [45,46] or a cell death mechanism induced by various anti-cancer agents [43,44,47,48,49,50]. Our results showed that cells with initiation of autophagy were more resistant to Tenovin-6, although the autophagy flux was blocked (Figure 3), suggesting a protective role of autophagy in these cells. Results of extensive studies in tumor cells in culture and in animal models suggested that autophagy can be induced by antitumor drugs and can be targeted to overcome chemotherapeutic resistance. Some early clinical results suggested that autophagy inhibition in combination with anticancer drugs seems to be safe and can fortify the efficacies of various anticancer therapies in glioblastoma, melanoma, and multiple myeloma [51,52]. Combined treatments with autophagy inhibitors such as hydroxychloroquine and chloroquine and other drugs showed promising results in some clinical cases [51]. Indeed, our results showed that combined treatment with both Tenovin-6 and chloroquine had synergistic inhibitory effects by inhibiting autophagy flux, inducing G1 arrest and apoptosis (Figure 4, Figure 5 and Figure 6). Hence, Tenovin-6 is an excellent candidate for combined use with autophagy inhibitor. Firstly, Tenovin-6 alone showed a promising anti-neoplastic effect in vitro or in vivo on various malignancies [6,7,8,11,12,13,53,54,55,56]; secondly, Tenovin-6 seems to be safe in mice, since no obvious adverse effect is reported so far [6,53,55,56,57]; thirdly, our results showed that Tenovin-6 initiated autophagy, which conferred a protective effect, in numerous cell lines; fourthly, this study indeed demonstrated that combined treatment with Tenovin-6 and chloroquine enhanced the cytotoxicity and synergistically induced apoptosis and G1 cell-cycle arrest in different GC cell lines compared to the treatment with either inhibitor alone (Figure 4 and Figure 5). Thus, dual treatment with Tenovin-6 and chloroquine could be a viable therapeutic approach for GC.

In summary, we reported that EBV-positive and -negative GC cell lines were sensitive to Tenovin-6 but with different sensitivities. Tenovin-6 induced different levels of apoptosis and phases of cell-cycle arrest, with some manifesting G1 and others showing S phase cell-cycle arrest depending on the genetic background of the cell lines (Table 1). The cytotoxic effect of Tenovin-6 was likely through activation of p53 or inhibition of autophagy flux. Initiation of autophagy had some protective effect on the cells; however, combined treatment with Tenovin-6 and autophagy inhibitor chloroquine increased the cytotoxic effect by inducing LC3B-II accumulation, and by enhancing apoptosis and cell-cycle arrest.

## 4. Materials and Methods

### 4.1. Reagents and Antibodies

The following regents were used in this study: Tenovin-6 (BSCC-37, Agave Pharm, Seattle, WA, USA) and chloroquine diphosphate (C6628, Sigma, St. Louis, MO, USA). Antibodies specific to the following proteins were used: β-actin (RM001V, Beijing Ray Antibody Biotech, Beijing, China), p53 (#2527, Cell Signaling Technology, Danvers, MA, USA), Ac-p53 (ab75754, Abcam, Cambridge, MA,USA), p-p53 (#9284, Cell Signaling Technology, Danvers, MA, USA), p21 (#2947, Cell Signaling Technology, Danvers, MA, USA), L3B (sc-376404, Santa Cruz, Dallas, TX, USA), and p62 (18420-1-AP, Proteintech Group, Inc., Chicago, IL, USA).

### 4.2. Cell Culture

AGS (American Type Culture Collection) is an Epstein–Barr virus (EBV)-negative cell line. AGS-EBV cells were obtained by infecting AGS cells with recombinant EBV M81. SNU-719, a gift from Dr. Kang, is a GC cell line containing native EBV genomes isolated from a GC patient. HGC-27, N87, SNU-1, and KATO-III are EBV-negative GC cell lines obtained from the Cell Bank of the Chinese Academy of Sciences. GC cell lines were cultured in Roswell Park Memorial Institute (RPMI)-1640 medium supplemented with 10% fetal bovine serum (FBS), penicillin–streptomycin solution, and l-glutamine at 37 °C and 5% CO_2_.

### 4.3. Western-Blotting

Cells were lysed in sample buffer (Gbcbio Technologies Inc., Guangzhou, China) supplemented with proteinase inhibitors (50×) and phosphatase inhibitors (50×). Protein lysates were resolved by SDS-PAGE and transferred onto a polyvinylidene fluoride (PVDF) membrane. After blocking with 5% skim milk, the membrane was probed with primary and secondary antibodies, and visualized using the enhanced chemiluminescence (ECL) system (Gbcbio Technologies Inc., Guangzhou, China).

### 4.4. Cell Cycle and Apoptosis Assays

To examine cell cycle, cells were trypsinized and fixed in 70% cold ethanol at −20 °C overnight. After treatment with 100 μg/mL RNase A, cells were stained with 50 μg/mL propidium iodide (PI) for 30 min at 37 °C and subjected to cell-cycle analysis. To examine apoptosis, cells were trypsinized without ethylenediaminetetraacetic acid (EDTA). Apoptotic cells were monitored using the Annexin V-Alexa Fluor 647/PI Apoptosis Detection Kit (Beijing 4A Biotech Co., Ltd., Beijing, China). Flow cytometry was performed on the NovoCyte Flow Cytometer (ACEC Biosciences. Inc., San Diego, CA, USA).

### 4.5. Colony Formation in Soft Agar

A total of 10^4^ cells suspended in 1 mL of 0.3% top agar (catalog no. A5431; Sigma-Aldrich, St. Louis, MO, USA) were plated onto one well of 0.5% base agar in six-well plates and maintained for 2–3 weeks. Colonies with a diameter of >50 μm were counted and photographed at 5× magnification using a microscope.

### 4.6. Statistical Analysis

Statistical analysis was performed using two-tailed t-tests, and statistical symbols *, **, and *** represent *p* values of <0.05, <0.01, and <0.001, respectively, while “NS” indicates “not significant”. Data are expressed as the mean ± SD of triplicate samples, and the reproducibility was confirmed in at least three independent experiments.

## 5. Conclusions

The current study provides experimental evidence demonstrating that Tenovin-6 has a promising anti-proliferative effect on GC cells in vitro. Furthermore, the combination of Tenovin-6 and chloroquine increased the cytotoxic effect by inducing LC3B-II accumulation, and by enhancing apoptosis and cell-cycle arrest. Treatment with Tenovin-6 alone or in combination with chloroquine could be a promising therapeutic approach for GC.

## Figures and Tables

**Table 1:** Summary of the effects of Tenovin-6 on gastric cancer cell lines.

Cell Line	Inhibition of Proliferation by Tenovin-6			Apoptosis (%) ^4^	Cell Cycle (%) ^4^			*TP53* Gene	Total p53	p-p53	Ac-p53	p21	LC3B	p62
	Con (μM) ^1^	Tm (h) ^2^	Inh (%) ^3^		G1	S	G2							
AGS	0.5	48	81.5	↑7.2 ± 4.4	↓13.8 ± 1.6	↑16.6 ± 1.9	↑2.7 ± 1.3	WT	↑	↑	↑	↑	UC	UC
AGS-EBV	0.5	48	88.3	↑8.6 ± 4.3	↓8.9 ± 3.1	↑29.7 ± 3.5	↓15.4 ± 2.5	WT	↑	↑	↑	↑	UC	UC
SNU-719	8.0	48	76.6	↑6.1 ± 0.6	↑28.1 ± 0.7	↓25.0 ± 0.3	↓3.0 ± 1.0	WT	↑	↑	↑	↑	↑	UC
HGC-27	1.0	72	86.2	↑13.3 ± 1.3	↓12.2 ± 1.3	↑21.9 ± 0.9	↓9.7 ± 0.8	Mut	ND	ND	ND	UC	↑	↑
N87	3.0	48	93.3	↑30.9 ± 3.0	↑22.8 ± 1.5	↓20.2 ± 2.8	↓2.6 ± 3.4	Mut	UC	UC	UC	UC	↑	↑
SNU-1	1.0	48	65.7	↑13.9 ± 0.9	↑30.3 ± 1.1	↓21.0 ± 1.4	↓9.3 ± 1.5	WT	↑	↑	↑	UC	↑	UC
KATO-III	1.0	48	44.8	↑8.6 ± 0.3	↑27.3 ± 0.3	↓29.4 ± 0.2	↑2.1 ± 0.3	Null	ND	ND	ND	UC	↑	UC

^1^ Concentration of Tenovin-6 used; ^2^ Tenovin-6 treatment time; ^3^ Inhibition of cell proliferation by Tenovin-6; ^4^ Percentages of changes of treated cells minus untreated cells with blue color denotes *p* < 0.05 and green color denotes *p* < 0.01; WT: wild-type *TP53* gene; Mut: mutated *TP53* gene; Null: null *TP53* gene; ND: not detected; UC: unchanged.

## Supplementary Materials

The following are available online at https://www.mdpi.com/2072-6694/12/2/365/s1, Figure S1: Original western blots for Figure 3 and Figure 6.
