# Supplementary material for: Heterogeneous Responses of Gastric Cancer Cell Lines to Tenovin-6 and Synergistic Effect with Chloroquine
Source: Cancers (Basel). 2020 Feb 5;12(2):365. doi: 10.3390/cancers12020365 (PMC7072542; doi:10.3390/cancers12020365)

Supplemental materials for Fig. 3-1

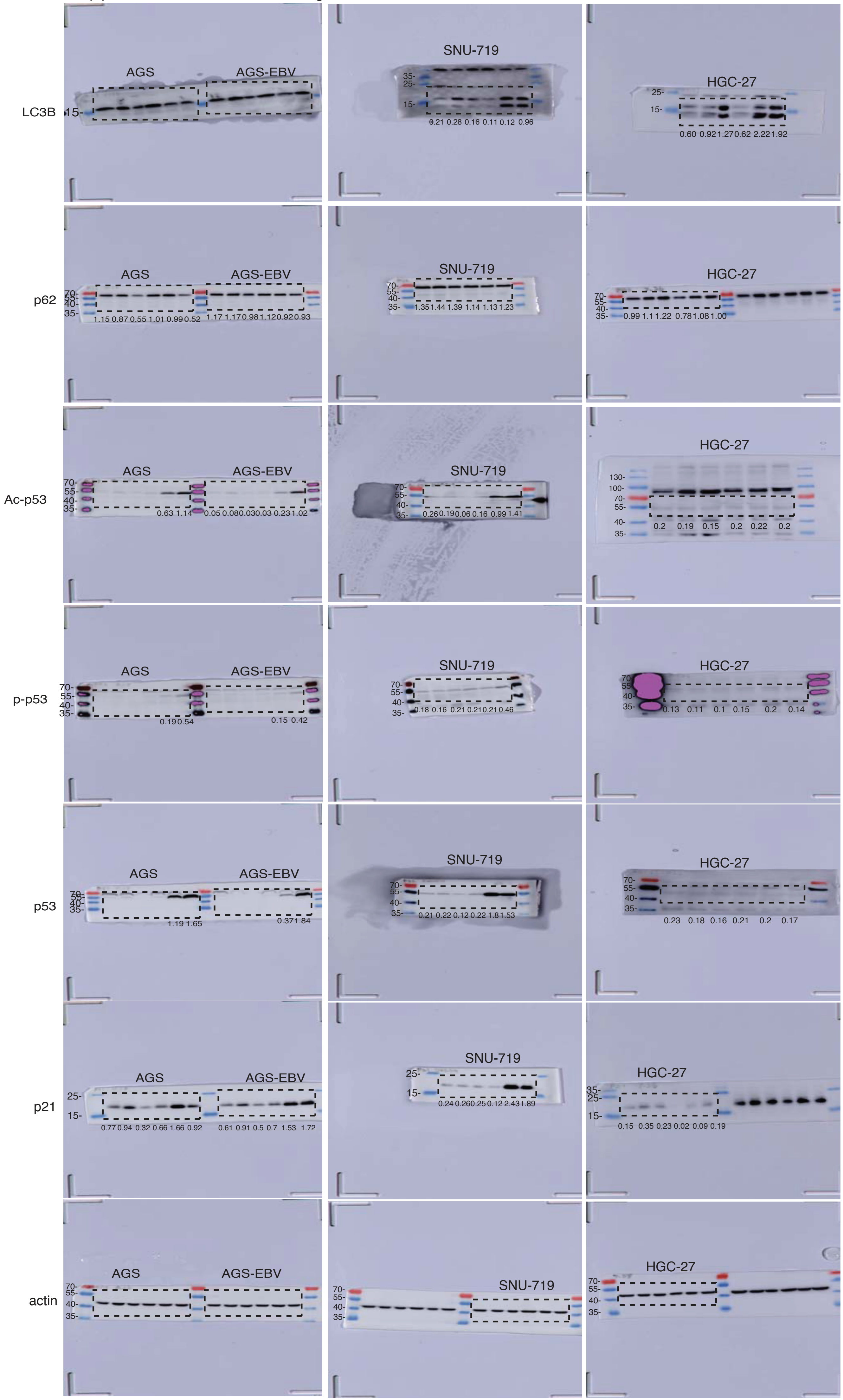

Supplemental materials for Fig. 3-2

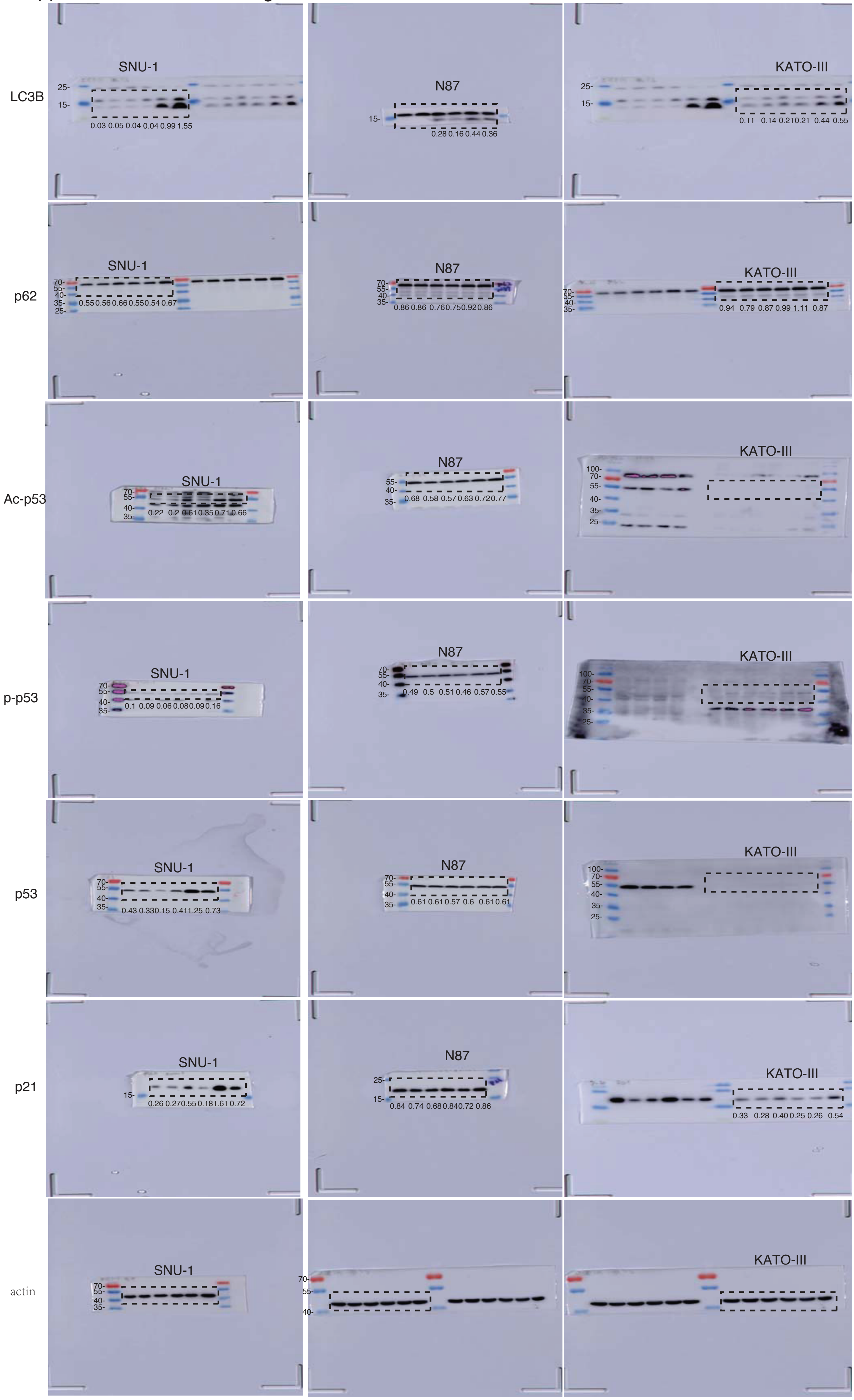

Supplemental materials for Fig. 6-1

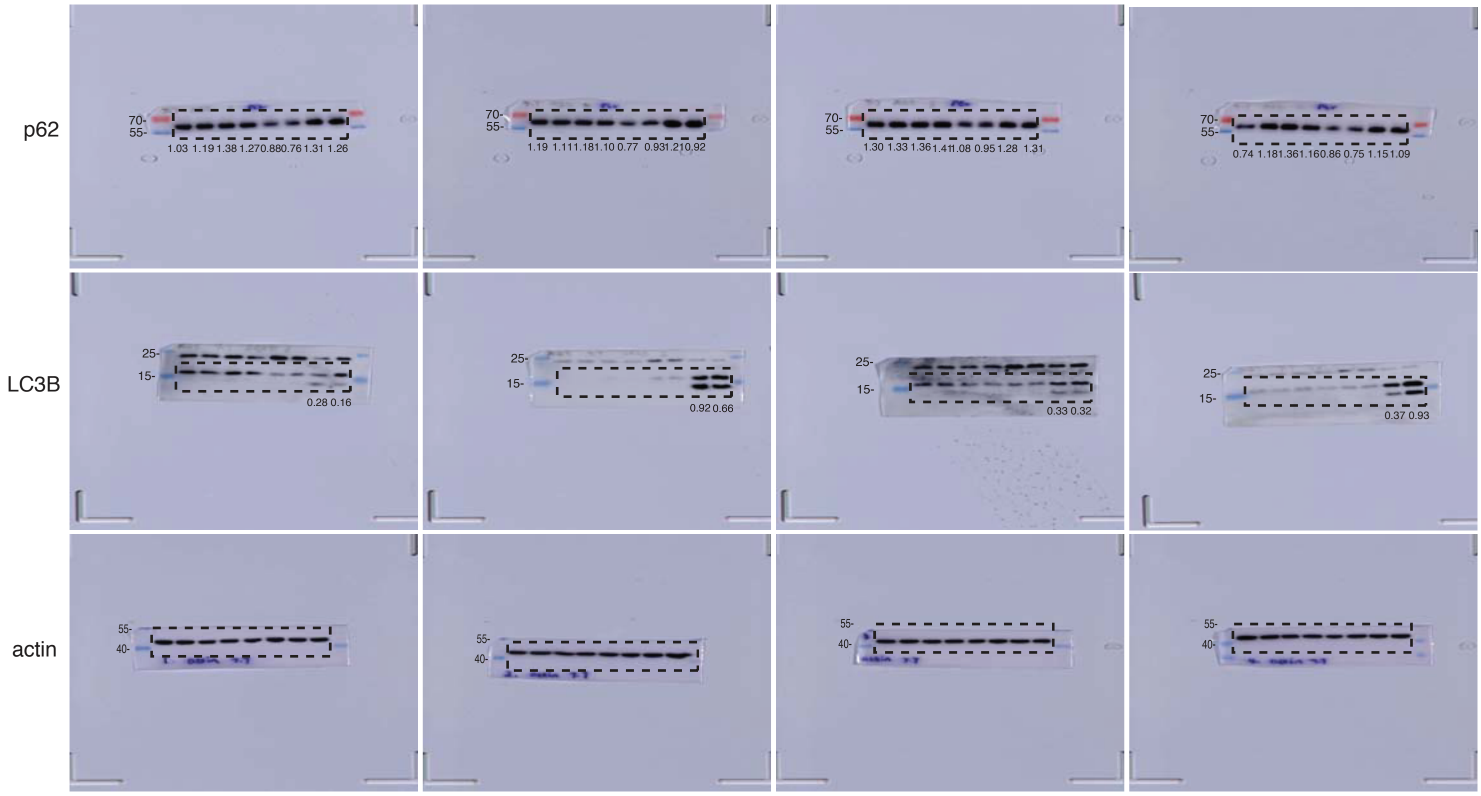

Fig 6B

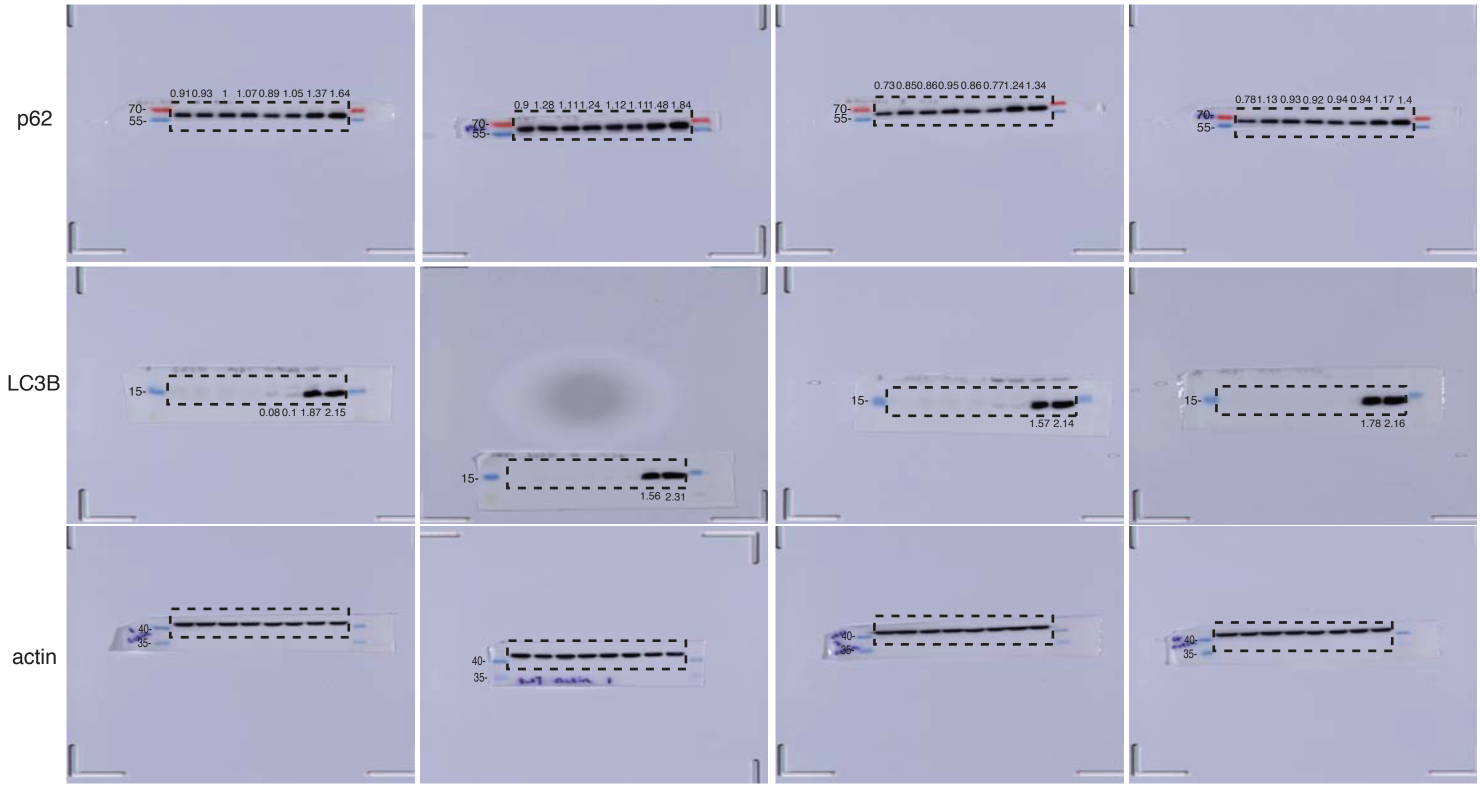

Supplemental materials for Fig. 6-2

p62

LC3B

actin

Fig 6D

p62

LC3B

actin

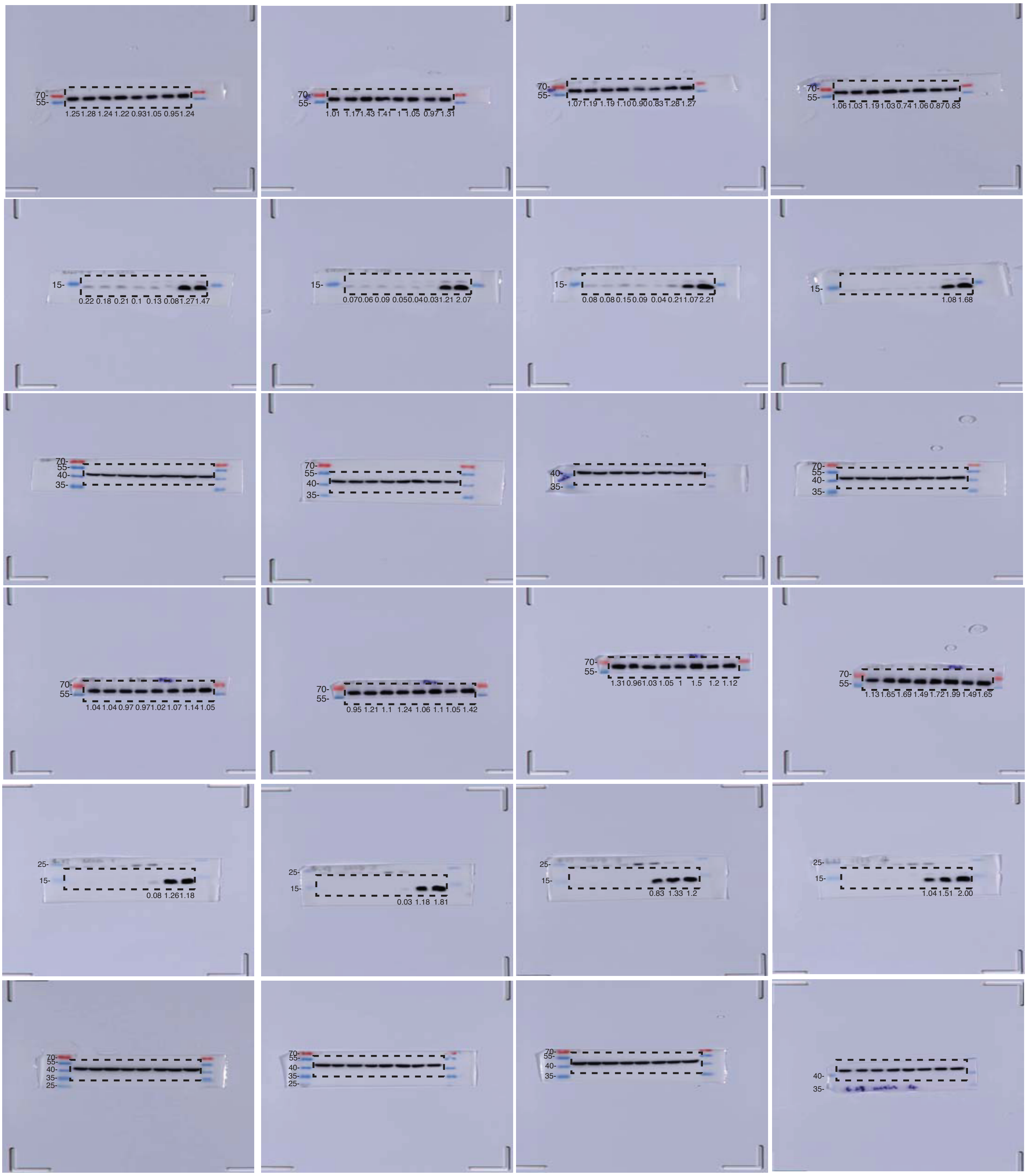

Supplement: Supplementary file 1 [file cancers-12-00365-s001.pdf]
